# COVID-19 Clinical Profile in Latin American Migrants Living in Spain: Does the Geographical Origin Matter?

**DOI:** 10.3390/jcm10225213

**Published:** 2021-11-09

**Authors:** Abiu Sempere, Fernando Salvador, Arnau Monforte, Júlia Sampol, Juan Espinosa-Pereiro, Marta Miarons, Pau Bosch-Nicolau, Alfredo Guillén-del-Castillo, Maria Luisa Aznar, Isabel Campos-Varela, Adrián Sánchez-Montalvá, Lina María Leguízamo-Martínez, Inés Oliveira, Andrés Antón, Benito Almirante

**Affiliations:** 1 Department of Infectious Diseases, Vall d’Hebron University Hospital, PROSICS Barcelona, 08035 Barcelona, Spain; abiusempere@gmail.com (A.S.); amonforte92@gmail.com (A.M.); macspinosa@gmail.com (J.E.-P.); pau.boschnicolau@gmail.com (P.B.-N.); maznarru@gmail.com (M.L.A.); adsanche@vhebron.net (A.S.-M.); inesoliveirasouto@gmail.com (I.O.); balmirante@vhebron.net (B.A.); 2 Department of Pneumology, Vall d’Hebron University Hospital, 08035 Barcelona, Spain; jusampol@gmail.com; 3 Department of Pharmacy, Vall d’Hebron University Hospital, 08035 Barcelona, Spain; miaronsm@gmail.com; 4 Department of Internal Medicine, Vall d’Hebron University Hospital, 08035 Barcelona, Spain; alguillen@vhebron.net; 5 Liver Unit, Vall d’Hebron Hospital Universitari, Vall d’Hebron Institut de Recerca (VHIR), Vall d’Hebron Barcelona Hospital Campus, Universitat Autònoma de Barcelona, 08035 Barcelona, Spain; icampos@vhebron.net; 6 Centro de Investigación Biomédica en Red de Enfermedades Hepáticas y Digestivas (CIBERehd), Instituto de Salud Carlos III, 28029 Madrid, Spain; 7 Department of Pharmacovigilance and Pharmacoepidemiology, Vall d’Hebron University Hospital, 08035 Barcelona, Spain; lina.leguizamo@vhir.org; 8 Department of Microbiology, Vall d’Hebron University Hospital, 08035 Barcelona, Spain; aanton@vhebron.net

**Keywords:** COVID-19, SARS-CoV-2, Latin America, severity

## Abstract

The aim of this study was to describe and compare the clinical characteristics of hospitalized patients with COVID-19 pneumonia according to their geographical origin. This is a retrospective case-control study of hospitalized patients with confirmed COVID-19 pneumonia treated at Vall d’Hebron University Hospital (Barcelona) during the first wave of the pandemic. Cases were defined as patients born in Latin America and controls were randomly selected among Spanish patients matched by age and gender. Demographic and clinical variables were collected, including comorbidities, symptoms, vital signs and analytical parameters, intensive care unit admission and outcome at 28 days after admission. Overall, 1080 hospitalized patients were registered: 774 (71.6%) from Spain, 142 (13.1%) from Latin America and the rest from other countries. Patients from Latin America were considered as cases and 558 Spanish patients were randomly selected as controls. Latin American patients had a higher proportion of anosmia, rhinorrhea and odynophagia, as well as higher mean levels of platelets and lower mean levels of ferritin than Spanish patients. No differences were found in oxygen requirement and mortality at 28 days after admission, but there was a higher proportion of ICU admissions (28.2% vs. 20.2%, *p* = 0.0310). An increased proportion of ICU admissions were found in patients from Latin America compared with native Spanish patients when adjusted by age and gender, with no significant differences in in-hospital mortality.

## 1. Introduction

At the end of 2019, China first reported a group of cases of severe acute respiratory syndrome by a new virus called SARS-CoV-2. Currently, it is a global pandemic and 172,956,039 cases have been reported and 3,726,466 people have died in total by 7 June 2021 [1]. The clinical presentation of COVID-19 is variable, ranging from asymptomatic presentations to flu-like symptoms or adult respiratory distress syndrome (ARDS). Respiratory complications typically appear about 7–10 days after the first symptom [2] causing significant morbidity and mortality [3,4,5].

Many risk factors have been described that predict the evolution throughout the hospitalization of these patients, such as being aged > 65 years, male, a high Charlson score and a respiratory rate upon their admission consistently associated with increased mortality [6,7,8,9,10]. Lately, some studies from the USA and Europe have suggested a possible association between the geographical origin of the patients, such as the Latin American or the Afro-American population, and the infection rate, need for hospitalization and death. Furthermore, studies of other respiratory infectious diseases, such as influenza, specifically H1N1 influenza, have also suggested links between geographical origin and worse outcomes, as well as the widespread nature of the COVID-19 pandemic [11,12,13]. All these results could be explained by a greater social vulnerability, access to health care, lack of testing for SARS-CoV-2 infection, higher virus exposure in essential-worker occupations and individual genetic factors [14,15,16,17,18,19,20,21,22]. Spain is fifth in the European Union regarding the highest rates of migration in the last 25 years, especially from Latin America, an ethnic group severely punished during the first wave of the COVID-19 pandemic [23,24].

The objectives of the study are (1) to describe the main clinical characteristics of a cohort of patients hospitalized with a diagnosis of pneumonia caused by SARS-CoV-2 and (2) to compare clinical variables and outcomes between Spanish natives and Latin American patients.

## 2. Methods

The Vall d’Hebron COVID-19 Prospective Cohort Study includes all consecutive adult patients (≥18 years old) who were hospitalized for COVID-19 at Vall d’Hebron University Hospital, a 1100-bed public tertiary care hospital in Barcelona (Spain). The hospital is the reference center of an area with a total population of 1,664,182 (1,305,095 citizens from Spain, 131,653 from the rest of Europe, 24,580 from Africa, 38,710 from North-Central America, 84,357 from South America and 79,787 Asia-Oceania) [25].

This is a retrospective case-control study in which all hospitalized patients treated at the hospital during the first wave (from 1 March to 30 April 2020) with laboratory-confirmed COVID-19 and radiologically confirmed pneumonia were included. A laboratory-confirmed case was defined as a patient with a real-time reverse-transcriptase polymerase chain reaction (RT-PCR) SARS-CoV-2-positive result in any respiratory sample (nasopharyngeal swab, sputum, bronchoalveolar lavage or aspirate, tracheal aspirate). Patients born in Latin American countries were defined as cases; controls were randomly selected from those Caucasian people born in Spain in a 1:4 ratio and matched by age and gender. The main exclusion criterion was the absence of pneumonia in the chest X-ray.

Overall, 1080 patients with confirmed COVID-19 pneumonia hospitalized during the study period were registered in the database: 774 (71.6%) from Spain, 142 (13.1%) from Latin America (LA), 43 (3.9%) from other countries and 121 (11.2%) cases without information regarding country of origin. From the 142 patients from LA, all of them fulfilled the inclusion criteria and were considered as cases. On the other hand, 558 Spanish patients were randomly selected as controls. Overall, 700 patients were included in this study.

Data were recorded in the Research Electronic Data Capture software (REDCap, Vanderbilt University). We collected variables including age, gender, country of origin, comorbidities (arterial hypertension, diabetes mellitus, chronic lung diseases, chronic heart diseases), behavioral factors, such as smoking or alcohol consumption, symptoms, vital signs and analytical parameters at admission, need of intensive care unit (ICU) admission, treatment received and outcome at 28 days after admission (discharge, still hospitalized or death).

Patients were treated according to the hospital’s clinical protocol available at that time. Tocilizumab was reserved for patients with respiratory failure and interleukin (IL)-6 levels > 40 pg/mL or D-dimer > 1500 ng/mL.

Continuous variables were expressed as mean and standard deviation or medians and interquartile range, as appropriate. Categorical variables were summarized as absolute numbers and percentages. As data are age and gender matched case-control, in order to compare differences, the usual contrast test cannot be used, as it does not take into account the dependency of case and control. A conditional univariable logistic regression was performed and the *p* value from the likelihood ratio test of the models with and without the covariate was calculated. In that sense, each case was compared with their corresponding controls. A type I error of 5% was considered. All analyses were carried out with STATA 15.1 (StataCorp, College Station, TX, USA).

Procedures were performed in accordance with the ethical standards laid down in the Declaration of Helsinki as revised in 2013, and the study protocol was approved by the Ethical Review Board of the Vall d’Hebron University Hospital (Barcelona, Spain). The Ethical Review Board agreed that informed consent was not necessary given the retrospective nature of the study and the anonymization of the information.

## 3. Results

As mentioned previously, 700 patients were selected for the study. From the 142 patients who were born in LA, their countries of origin were as follows: Ecuador: 23.9% (34/142), 29 (20.4%) from Perú, 19 (13.4%) from Bolivia, 17 (12.0%) from Honduras, 15 (10.7%) from Colombia, 9 (6.3%) from Venezuela, 4 (2.8%) from Dominican Republic, 3 (2.1%) from Brazil, 3 (2.1%) from Chile, 3 (2.1%) from Cuba, 2 (1.4%) from El Salvador, 2 (1.4%) from Paraguay and 2 (1.4%) from Uruguay. From the overall study population (700 patients), the epidemiological and clinical characteristics of patients are summarized in Table 1, Table 2, Table 3 and Table 4. The mean age in the study population was 47.67 (SD 12.6) years, and 382 (54.6%) were men. The most frequent comorbidities were arterial hypertension (21.1%), lung diseases including asthma and chronic obstructive pulmonary disease (12%), diabetes mellitus (9.4%) and smoking (6.9%). Regarding the symptoms at admission, the majority had fever (90.9%), cough (79.1%) and dyspnea (52.7%), followed by arthromyalgia (31.9%), gastrointestinal symptoms (diarrhea 28.7%, nausea 7% and vomiting 8.1%), anosmia (8.4%) and rhinorrhea (3.6%). Systemic inflammation biomarkers were increased with Dimer-D mean levels of 1342.95 (10–2695.97) ng/mL, LDH of 369.17 (356.79–381.54) UI/L, C-reactive protein of 11.16 (10.27–27.42) mg/dL, ferritine of 742.15 (656–827.58) pg/mL and IL-6 of 141.53 (47.84–235.21) pg/mL. Overall, 150 (21.7%) patients needed ICU admission, and 8 (2.1%) patients needed hospital readmission. After 28 days of admission, 102 (14.6%) patients were still hospitalized, 576 (82.3%) patients were discharged and 22 (3.1%) died.

When comparing clinical symptoms at admission between both groups, we observed that patients from LA had a higher proportion of anosmia (14.8% vs. 6.8%, *p* = 0.0026), rhinorrhea (7% vs. 2.7%, *p* = 0.0144) and odynophagia (9.9% vs. 5%, *p* = 0.0312) than Spanish patients. Regarding analytical parameters at admission, patients from LA had higher mean levels of platelets (241.41 × 10^9^/L vs. 211.36 × 10^9^/L, *p* = 0.0025) and lower mean levels of ferritin (550.46 ng/mL vs. 795.6 ng/mL, *p* = 0.0384) than Spanish patients. When comparing clinical outcomes at 28 days after admission, patients from Latin America showed a higher proportion of ICU admissions (28.2% vs. 20.2%, *p* = 0.0310) than Spanish patients. However, we did not find any difference between both groups regarding oxygen requirement and mortality.

## 4. Discussion

People from racial and ethnic minority groups were disproportionately affected by the SARS-CoV-2 pandemic in high-income countries. Some studies performed during the first wave of the pandemic have shown a higher risk of infection and worse outcomes among some migrant population groups [26,27]. In the present study, we compared clinical characteristics and outcomes of adult hospitalized patients with SARS-CoV-2 pneumonia, observing a higher risk of ICU admission among Latin American patients compared with Spanish patients after adjusting by age and gender. Although the proportion of the Latin American population at that time in Barcelona was 5.06% [25], the proportion observed in the first wave in our hospital was 13.1% (142/1080), which may suggest a certain susceptibility, likely because of the social determinants of health, jobs with greater exposure, family overcrowding or even genetic factors [28].

In the present case-control study, a higher prevalence of certain cardiovascular risk factors was observed in Spanish natives, such as arterial hypertension, heart failure and smoking, although without reaching statistical significance, and contrary to what was observed in an American series [29]. This could be partly explained by the concept of the healthy migrant effect [30,31] that indicates migrants often have a better health status than the remaining population in the native country, but also compared with the majority in the host country, especially during the first 5–10 years after migration.

A similar BMI was observed in both groups (29.3 vs. 30.4), when adjusted for age and gender, a degree that is considered pre-obesity [32], which could be dismissed as a determining factor in the evolution of patients of this study, contrary to what the literature shows [33,34,35], since it is considered an essential point in the creation of venous thrombosis [36,37], a relevant factor for the initiation of diffuse alveolar damage consistent with ARDS in the pathology of SARS-CoV-2 [38,39,40,41].

Regarding the clinical manifestations, we found a higher proportion of arthromyalgia, anosmia and cacosmia among Latin American patients, which could suggest a different pathophysiological response of the host against the virus. Related to anosmia, it has been hypothesized that SARS-CoV-2 has neurotropic characteristics, being able to invade peripheral nerve terminals and enter the central nervous system through trans-synaptic pathways [42,43]. It is known that the supporting cells of the olfactory epithelium express ACE-2, where the SARS-CoV-2 may bind, causing in this way anosmia and/or headache [44]. This explanation may warrant a different distribution of the ACE2 receptors in LA patients. Some analytical markers of inflammation have been associated with a worse evolution of the disease [45,46,47]. Although some differences were found regarding the mean value of platelets and ferritin among both groups in our study, we did not find any difference regarding the levels of IL-6, CRP and D-dimer, which are the most outcome-related biomarkers described.

The study showed a higher percentage of patients requiring ICU admission among Latin American patients compared with the Spanish population. It was in accordance with the oxygen requirements during admission (higher in the Latin American group), although it did not reach statistical significance. Moreover, although the need for ICU admission was higher, no differences in mortality were observed. These results could be explained by transient worsening in the clinical symptoms (mostly respiratory) due to an increase in systemic inflammation, which are sufficient for ICU admission needs, but not too severe for increasing the risk of death. Genetic factors that have not been studied yet but with a relevant role in pathophysiology might have also contributed [48,49].

It is important to note that current therapeutic options for COVID-19 pneumonia include the use of corticosteroids and other immunomodulators (such as tocilizumab or baricitinib), drugs that can increase the risk of reactivation of latent infections, including those endemic in geographical areas where migrants come from, such as the strongyloidiasis [50,51]. This reinforces the importance of screening for neglected latent infections in the migrant population, especially in those at risk of immunosuppression.

This study has some limitations given its retrospective nature. First, especially during the first wave of the pandemic, COVID-19 management protocol amendments or modifications were periodically performed. However, the clinical protocols for COVID-19 management used in this study were the same between Spanish natives and Latin Americans. Second, because of operational limitations during an outbreak, some patient’s information is missing, but the sample size of the study may have allowed obtaining comprehensive results. Third, we included patients admitted to the hospital due to COVID-19; therefore, even if we detected an increased rate of ICU admissions in LA, this precludes us from reaching conclusions regarding community transmission in this population group.

In summary, we observed an increased rate of ICU admissions among hospitalized adult patients with COVID-19 pneumonia from Latin America compared with native Spanish patients. Furthermore, there was a higher rate of arthromyalgia, cacosmia and anosmia observed in patients from Latin America. These differences could be explained by socio-economic factors or still unknown genetic factors, so further investigations are needed. Understanding ethnic disparities can help so as to create health prevention strategies.

## Figures and Tables

**Table 1:** Epidemiology and comorbidities in hospitalized patients with COVID-19 pneumonia according to the geographical area of origin.

Epidemiology and Comorbidities	Study Population (*n* = 700)	Spanish Controls (*n* = 558)	Latin American (*n* = 142)	Univariate Analysis (*p*-Value)
Age, years	47.67 (SD 12.66)	47.81 (SD 12.61)	47.12 (SD 12.87)	-
Gender, male	382 (54.6%)	304 (54.5%)	78 (54.9%)	-
Smoking	48 (6.9%)	43 (7.8%)	5 (3.5%)	*p* = 0.080
Alcohol consumption	28 (4.1%)	21 (3.8%)	7 (4.9%)	*p* = 0.514
BMI	29.53 (SD 5.22)	29.30 (SD 5.13)	30.49 (SD 5.52)	*p* = 0.259
Arterial hypertension	147 (21.1%)	122 (21.9%)	25 (17.7%)	*p* = 0.292
Chronic heart diseases	8 (1.1%)	7 (1.3%)	1 (0.7%)	*p* = 0.697
Diabetes mellitus	65 (9.4%)	49 (8.9%)	16 (11.4%)	*p* = 0.273
Chronic lung diseases	84 (12.0%)	71 (12.7%)	13 (9.2%)	*p* = 0.264
Asthma	44 (6.3%)	40 (7.2%)	4 (2.8%)	*p* = 0.065
COPD	15 (2.1%)	14 (2.4%)	1 (0.7%)	*p* = 0.211
Interstitial pneumopathy	3 (0.4%)	3 (0.5%)	0 (0%)	*p* = 1
Bronchial hyperresponsiveness	2 (0.3%)	0 (0%)	2 (1.4%)	*p* = 1
Other pneumopathies	13 (1.9%)	9 (1.6%)	4 (2.8%)	*p* = 0.3383

BMI: body mass index; COPD: chronic obstructive pulmonary disease. Note: Data are reported as number (%) of patients or mean (SD).

**Table 2:** Clinical symptoms at admission in hospitalized patients with COVID-19 pneumonia according to the geographical area of origin.

Clinical Symptoms	Study Population (*n* = 700)	Spanish Controls (*n* = 558)	Latin American (*n* = 142)	Univariate Analysis (*p*-Value)
Fever	636 (90.9%)	507 (90.9%)	129 (90.8%)	*p* = 0.960
Cough	554 (79.1%)	437 (78.3%)	117 (82.4%)	*p* = 0.301
Dyspnea	369 (52.7%)	291 (52.2%)	78 (54.9%)	*p* = 0.481
Malaise	276 (39.4%)	211 (37.8%)	65 (45.8%)	*p* = 0.106
Arthromyalgia	223 (31.9%)	170 (30.5%)	53 (37.3%)	*p* = 0.121
Diarrhea	201 (28.7%)	165 (29.6%)	36 (25.4%)	*p* = 0.362
Expectoration	126 (18.0%)	99 (17.7%)	27 (19%)	*p* = 0.686
Anosmia	59 (8.4%)	38 (6.8%)	21 (14.8%)	*p* = 0.002
Pleuritic chest pain	138 (19.7%)	117 (21%)	21 (14.8%)	*p* = 0.068
Vomits	57 (8.1%)	43 (7.7%)	14 (9.9%)	*p* = 0.402
Nausea	49 (7.0%)	38 (6.8%)	11 (7.7%)	*p* = 0.706
Rhinorrhea	25 (3.6%)	15 (2.7%)	10 (7%)	*p* = 0.014
Odynophagia	42 (6.0%)	28 (5.0%)	14 (9.9%)	*p* = 0.031
Nasal congestion	16 (2.3%)	13 (2.3%)	3 (2.1%)	*p* = 0.900
Hemoptysis	10 (1.4%)	8 (1.4%)	2 (1.4%)	*p* = 1
Weight loss	5 (0.7%)	4 (0.7%)	1 (0.7%)	*p* = 1
Night sweats	5 (0.7%)	3 (0.5%)	2 (1.4%)	*p* = 0.282
Cacosmia	2 (0.3%)	1 (0.2%)	1 (0.7%)	*p* = 0.327

Note: Data are reported as number (%) of patients or mean (SD).

**Table 3:** Analytical parameters at admission in hospitalized patients with COVID-19 according to the country of origin.

Analytical Parameters	Study Population (*n* = 700)	Spanish Controls (*n* = 558)	Latin American (*n* = 142)	Univariate Analysis (*p*-Value)
Hemoglobin (g/dL)	13.49 (SD 1.76)	13.51 (SD 1.75)	13.42 (SD 1.80)	*p* = 0.631
Leukocytes (×10^9^/L)	8.52 (SD 18.66)	8.46 (SD 20.33)	8.74 (SD 10.48)	*p* = 0.823
Lymphocytes (%)	18.75 (SD 10.22)	18.92 (SD 10.53)	18.13 (SD 9.00)	*p* = 0.307
Platelets (×10^9^/L)	217.78 (SD 94.71)	211.36 (SD 91.88)	241.41(SD 101.35)	*p* = 0.002
D-dimer (ng/mL)	1342.95 (SD 16005.72)	1511.67 (SD 18042.90)	758.71 (SD 4033.36)	*p* = 0.700
Creatinine (mg/dL)	0.88 (SD 0.57)	0.89 (SD 0.58)	0.85 (SD 0.53)	*p* = 0.401
AST (UI/L)	51.55 (SD 39.26)	52.48 (SD 41.50)	48.11 (SD 29.43)	*p* = 0.260
ALT (UI/L)	44.25 (SD 44.45)	43.27 (SD 46.50)	47.86 (SD 35.92)	*p* = 0.307
LDH (UI/L)	369.17 (SD 133.26)	374.53 (SD 132.16)	349.5 (SD 136.12)	*p* = 0.171
CRP (mg/dL)	11.16 (SD 10.40)	11.18 (SD 10.72)	11.10 (SD 9.22)	*p* = 0.721
Ferritin (ng/mL)	742.15 (SD 949.53)	795.6 (SD 1038.57)	550.46 (SD 474.39)	*p* = 0.038
IL-6 (pg/mL)	141.53 (SD 1115.43)	152.79 (SD 1261.51)	103.5 (SD 270.99)	*p* = 0.607

AST: aspartate aminotransferase; ALT: alanine aminotransferase; LDH: lactate dehydrogenase; CRP: C-reactive protein. Note: Data are reported as number (%) of patients or mean (SD).

**Table 4:** Clinical outcomes in hospitalized patients with COVID-19 pneumonia according to the geographical area of origin.

Outcomes	Study Population (*n* = 700)	Spanish Controls (*n* = 558)	Latin American (*n* = 142)	Univariate Analysis (*p*-Value)
ICU admission	150 (21.7%)	110 (20.2%)	40 (28.2%)	*p* = 0.031
Oxygen requirement				
No oxygen requirement	310 (44.3%)	254 (45.5%)	56 (39.4%)	*p* = 0.490
Low-flow oxygen ^a^	239 (34.1%)	188 (33.7%)	51 (35.9%)	
High-flow oxygen ^b^	43 (6.1%)	33 (5.9%)	10 (7%)	
IMV	108 (15.4%)	83 (14.9%)	25 (17.6%)	
Outcome at 28 days				
Discharge	576 (82.3%)	453 (81.2%)	123 (86.6%)	*p* = 0.726
Hospitalization ^c^	102 (14.6%)	83 (14.9%)	19 (13.4%)	
Death	22 (3.1%)	22 (3.9%)	0 (0%)	
Readmission	8 (2.1%)	7 (2.2%)	1 (1.5%)	*p* = 0.771

ICU: intensive care unit; IMV: invasive mechanical ventilation. ^a^ Low-flow oxygen includes oxygen mask and nasal cannulae; ^b^ High-flow oxygen includes high-flow nasal cannulae and non-invasive mechanical ventilation; ^c^ Hospitalization includes patients who are still hospitalized or referred to other centers. Note: Data are reported as number (%) of patients or mean (SD).

## Data Availability

All data generated or analyzed during this study are included in this published article.
